# Supplementary material for: Prediction of Non-Response to Neoadjuvant Chemoradiotherapy in Esophageal Cancer Patients with 18F-FDG PET Radiomics Based Machine Learning Classification
Source: Diagnostics (Basel). 2022 Apr 24;12(5):1070. doi: 10.3390/diagnostics12051070 (PMC9139915; doi:10.3390/diagnostics12051070)
Supplement: Supplementary file 1 [file diagnostics-12-01070-s001.zip › diagnostics-1650580-supplementary.pdf]

Supplementary Table S1

Radiomic Feature	Reason to exclude
joint_maximum_3D_comb	Not reproducable
inverse_difference_normalised_3D_comb	Not reproducable
inverse_difference_moment_normalised_3D_comb	Not reproducable
cluster_shade_3D_comb	Not reproducable
cluster_prominence_3D_comb	Not reproducable
low_dependence_low_grey_level_emphasis_3D	Not reproducable
dependence_count_energy_3D	Not reproducable
small_zone_low_grey_level_emphasis_3D	Not reproducable
small_zone_high_grey_level_emphasis_3D	Not reproducable
large_zone_low_grey_level_emphasis_3D	Not reproducable
grey_level_non_uniformity_normalised_GLSZM_3D	Not reproducable
grey_level_variance_GLSZM_3D	Not reproducable
small_distance_high_grey_level_emphasis_3D	Not reproducable
large_distance_low_grey_level_emphasis_3D	Not reproducable
grey_level_non_uniformity_normalised_DZM_3D	Not reproducable
grey_level_variance_3D	Not reproducable
zone_size_entropy_DZM_3D	Not reproducable
centre_of_mass_shift	Not reproducable
quartile_coefficient_of_dispersion	Not reproducable
V10	Not reproducable
DV1090	Not reproducable
dependence_count_percentage_3D'	Not reproducable
approximate volume	Redundant
surface area	Redundant
maximum 3D diameter	Redundant
major axis length	Redundant
minor axis length	Redundant
least axis length	Redundant
elongation	Redundant
flatness	Redundant
volume density AABB	Redundant
Morans I index	Redundant
Gearys C measure	Redundant
skewness	Redundant
P90	Redundant
energy intensity	Redundant
root mean square	Redundant
V90	Redundant
I10	Redundant
AUIVHC	Redundant
joint entropy 3D comb	Redundant
difference average 3D comb	Redundant
difference variance 3D comb	Redundant

difference entropy 3D comb	Redundant
sum average 3D comb	Redundant
sum variance 3D comb	Redundant
sum entropy 3D comb	Redundant
contrast GLCM 3D comb	Redundant
inverse difference 3D comb	Redundant
inverse variance 3D comb	Redundant
autocorrelation 3D comb	Redundant
cluster tendency 3D comb	Redundant
first measure of information correlation 3D comb	Redundant
second measure of information correlation 3D comb	Redundant
short runs emphasis 3D comb	Redundant
long runs emphasis 3D comb	Redundant
low grey level run emphasis 3D comb	Redundant
high grey level run emphasis 3D comb	Redundant
short run low grey level emphasis 3D comb	Redundant
short run high grey level emphasis 3D comb	Redundant
long run low grey level emphasis 3D comb	Redundant
long run high grey level emphasis 3D comb	Redundant
grey level non uniformity normalised GLRLM 3D comb	Redundant
run length non uniformity normalised 3D comb	Redundant
grey level variance GLRLM 3D comb	Redundant
run entropy 3D comb	Redundant
large zone emphasis 3D	Redundant
low grey level zone emphasis 3D	Redundant
high grey level zone emphasis 3D	Redundant
grey level non uniformity GLSZM 3D	Redundant
zone percentage 3D	Redundant
zone size variance 3D	Redundant
zone size entropy 3D	Redundant
small distance emphasis 3D	Redundant
small distance low grey level emphasis 3D	Redundant
grey level variance DZM 3D	Redundant
zone size variance DZM 3D	Redundant
busyness 3D	Redundant
texture strength 3D	Redundant
high dependence emphasis 3D	Redundant
low grey level count emphasis 3D	Redundant
low dependence high grey level emphasis 3D	Redundant
high dependence low grey level emphasis 3D	Redundant
high dependence high grey level emphasis 3D	Redundant
dependence count non uniformity 3D	Redundant
dependence count variance 3D	Redundant
dependence count entropy 3D	Redundant

Supplementary table S1 displays the excluded vendor dependent and redundant features. Abbreviations: GLSZM = gray level size zone matrix, DZM = zone distance entropy, GLRLM = grey-level run length matrix.
